# Fabrication of Green Diatomite/Chitosan-Based Hybrid Foams with Dye Sorption Capacity

**DOI:** 10.3390/ma13173760

**Published:** 2020-08-25

**Authors:** Barbara Galzerano, Carmen I. Cabello, Mercedes Muñoz, Giovanna G. Buonocore, Paolo Aprea, Barbara Liguori, Letizia Verdolotti

**Affiliations:** 1 Institute of Polymers, Composite and Biomaterials, National Research Council, P.le Enrico Fermi, Portici, 80055 Naples, Italy; barbara.galzerano@unina.it (B.G.); giovannagiuliana.buonocore@cnr.it (G.G.B.); letizia.verdolotti@cnr.it (L.V.); 2 ACLabs—Applied Chemistry Labs, Department of Chemical, Materials and Industrial Engineering, University of Naples Federico II, Piazzale V. Tecchio 80, 80125 Naples, Italy; paolo.aprea@unina.it; 3 “Centro de Investigacion y Desarollo en Ciencias Aplicadas Dr. J. J. Ronco” (CINDECA-CONICET-CIC-UNLP), Calle 47 N 257, 1900 La Plata, Argentine; cicabello.cabello@gmail.com (C.I.C.); mechemunioz@gmail.com (M.M.)

**Keywords:** diatomite, chitosan, sustainable hybrid foams, dye sorption

## Abstract

The latest tendency of the scientific community regards the development of different classes of green materials able to solve pollution problems caused by industrial and human activity. In this paper, chitosan and diatomite were used to produce a broad-spectrum hybrid adsorbent, either in powder or in monolithic form for environmental pollutant removal. Diatomite–chitosan-based powders and porous diatomite–chitosan hybrids were prepared and characterized by chemical-physical, thermal and morphological analysis. Moreover, their adsorbent capacity towards anionic dye (Indigo Carmine) was also evaluated. Obtained data showed that chitosan improves the adsorption capacity of both systems, increasing the uptake of dye in both diatomite–chitosan systems.

## 1. Introduction

The impact of toxic or hazardous compounds, such as heavy metals and dyes, on the environment is a huge problem, because they can alter several ecosystems by influencing the vital balance and the life mechanics of all living species [1]. Various mechanisms for metal immobilization have been proposed: adsorption [2], dissolution [3], redox or complexation reactions [4]. The environmental impact of these chemicals depends on whether they are dissolved, and therefore transported with a water mass, or adsorbed, and hence capable of settling out of solution in localized areas. Large-scale pollution caused by industrial, agricultural and municipal activities generates wastewater, which contains many toxic compounds [5]. For this reason, scientific and industrial communities are putting great effort into creating sustainable and multifunctional absorbent materials capable to remove or absorb toxic materials [6,7,8,9]. In this respect, the development of multifunctional porous materials based on natural compounds such as Diatomite [10] and chitosan [11] is a very interesting approach [12]. Diatomite is a fossil/mineral material with a large surface area and a great number of channels. Due to its intrinsic nanoporosity, a large adsorptive capacity can be observed; furthermore, it has many active groups and negative charge [13], and it produces no secondary pollution [14]. Chitosan is a low-cost natural molecule obtained from deacetylation of chitin (polysaccharide found in the exoskeleton of crustaceans and insects). Thanks to the high content of active functional groups such as amino and hydroxyl groups, it has drawn particular attention as a potential effective sorbent material, mainly for heterogeneous catalysis [15], drug delivery [16] and tissue engineering [17]. Among these applications, chitosan has received great interest as adsorbents for wastewater treatment [18,19,20,21]. On the other hand, in the recent literature, some papers deal with the combined use of both diatomite and chitosan to produce adsorbents for environmental pollutant removal, with the aim to evaluate their synergistic adsorption properties [22,23,24,25,26,27]. Herein, a chitosan-modified diatomite was synthesized with the aim to produce a novel, broad spectrum hybrid adsorbent. The combination of several and innovative manufacturing processes [9] allowed one to obtain adsorbent materials either in powder or in monolithic form (a ceramic foam from a natural source) with original, unique and tunable properties. For this purpose, chitosan-modified diatomite and porous diatomite–chitosan hybrids were produced and characterized by chemical-physical, thermal and morphological analysis. Indigo Carmine (IC), the most important organic dye, due to its extensive use in textile and other industries such as paper, plastic, leather, food, cosmetics and printing [28], was selected as a model dye to verify the suitability of the produced materials for environmental remediation application. Accordingly, its adsorbent capacity towards anionic dye (Indigo Carmine) was evaluated.

## 2. Materials and Methods

### 2.1. Materials

The Diatomite from “Antofagasta de la Sierra” (Catamarca, Argentine) was provided by the company CRYDON S.A. Diatomaceous earths are fossil siliceous materials, originating from unicellular organisms, whose agglutination results in amorphous siliceous rocks. According to the study of Maidana and Seeligmann [29], in our sample the majority observed forms could correspond to the so-called “Navicula parinacota and Planothidium chilense”. The particle size of the material was between 63 and 32 microns. Textural analysis was performed by N_2_ adsorption–desorption at 77 K using a Micromeritics ASAP 2020 analyzer (Norcross, GA, USA). The samples were preheated under vacuum in two steps of 1 h at 100 °C and 1 h at 200 °C. Data obtained are: Brunauer–Emmett–Teller (BET) specific surface area: 6.2859 ± 0.0273 m^2^/g, total pore volume: 0.023037 cm^3^/g.

Chitosan is a commercial product provided by Parafarm, Argentina (Mw: 273 kDa—DD: 95.2%) [30].

A commercial organic surfactant, Darafill 200, was supplied by GCP Applied Technologies (Cambridge, MA, USA). Si metal powder, Acetic acid, Na_2_SiF_6_ and Indigo Carmine anionic dye were all purchased by Sigma-Aldrich (St. Louis, MO, USA).

### 2.2. Preparation of Porous Diatomite–Chitosan Materials

A chitosan-modified diatomite was produced. Accordingly, chitosan powder (namely, C) was dissolved in a 4% *(wt/v)* of acetic acid solution at room temperature until complete dissolution, producing a light-yellow viscous solution. Subsequently, diatomaceous earth (namely, D) was added to chitosan solution and stirred until a homogeneous mixture was obtained. After that, diatomite chitosan-based powders (DC) were washed several times with distilled water, then dried at 100 °C for 3 h, grounded and sieved. Three different diatomite–chitosan-based powders at 20, 40 and 60 wt% of chitosan with respect to the amount of diatomite were prepared (namely, DC20, DC40 and DC60). The chitosan-modified diatomite powders were used to prepare porous diatomite–chitosan hybrids (namely, PDCs: PDC20, PDC40 and PDC60) according to the procedure described [9,31,32], in which sodium silicate (namely, SS) was used as crosslinker polymer in presence of suitable amount of Na_2_SiF_6_ used as catalyst (8.65 wt%). To produce porosity, two kinds of blowing agents were added in the aforementioned mixture, for instance, a “whipped” organic surfactant (meringue type foam [9,31]) produced by using a UltraTurrax disperser mixer (IKA-Werke GmbH & Co., Staufen, Germany (@1200 rpm) and Si metal powder that in alkaline solution through a redox reaction produce H_2_ gas [9,31].

Pristine porous diatomite-based material (PD, without chitosan) was prepared for the sake of comparison.

### 2.3. Characterizations

The chemical properties of C, D, DCs and PDCs samples were assessed by Fourier-transform infrared spectroscopy (FTIR) investigation [31] through FTIR spectrometer (Bruker IFS 66 FT-IR equipment, Bruker Optik GmbH, Ettlingen, Germany) by using KBr pellets in the range 4000–400 cm^−1^, and 64 scans were acquired with a resolution of 4 cm^−1^. The spectral region ranging from 1800 to 1500 cm^−1^ for selected samples (C and DC60) was deconvoluted by using OriginPro 8.0 software, OriginLab Corp., Northampton, MA, USA (Lorentzian fitting). The scanning electron microscopy (SEM, FEI ESEM Quanta 200(Hillsboro, OR, USA, acceleration voltage of 20 or 25 kV) was used to evaluate the morphological structure of PDCs. Furthermore, thermal degradation behavior of PDCs was investigated by means of thermogravimetric analysis (TG-DTA) (Netzsch, model 409ST Luxx, Selb, Germany), heating rate of 10 °C/min, under nitrogen atmosphere. The samples were heated on platinum pans from 25 to 1000 °C with heating rate of 10 °C/min. Furthermore, water absorption and porosity tests were assessed on PDCs samples (UNI 11060-03 and UNI EN 1936-01). Accordingly, each specimen was dried at 105 °C ± 5 °C until the mass became stable, and then it was brought in a chamber and immersed in water. By means of a vacuum pump, air was removed from the chamber for 30 min, and then it was filled with water until the specimen was covered. After 2 h, the chamber was returned to atmospheric pressure and PD and PCD60 samples were removed, shaken to remove excess of water, dried and weighted, to evaluate the amount of water absorbed (WA) and the total porosity of the materials (open and closed pores). Preliminary adsorption tests were carried out on DCs powders and PDCs hybrids using as dye Indigo Carmine (IC) solutions with a concentration of 70 mg/L. The dye-removal process was assessed by bringing in contact 125 mg of each sample with 50 mL of IC solution at room temperature and stirring at 500 rpm for 24 h. After that, the dye concentration was determined by using a spectrophotometer at the characteristic IC’s absorbance wavelength of ~610 nm. Using the following equations, the ion removal percentage and uptake capacity was calculated as follows:

(1) $$Removal\;\%=\frac{\left(C_{0}-C_{e}\right)}{C_{0}}\times 100$$

(2) $$q_{e}=\frac{\left(C_{0}-C_{e}\right)V}{m}$$

where *C*_0_ (mg/L) and *C_e_* (mg/L) are the initial and equilibrium IC concentrations, respectively; *q_e_* (mg/g) is the uptake capacity of IC by the adsorbent at equilibrium time; *V* (L) is the initial volume of solution; and *m* (g) is the mass of adsorbents.

## 3. Results and Discussion

**Figure 1:**
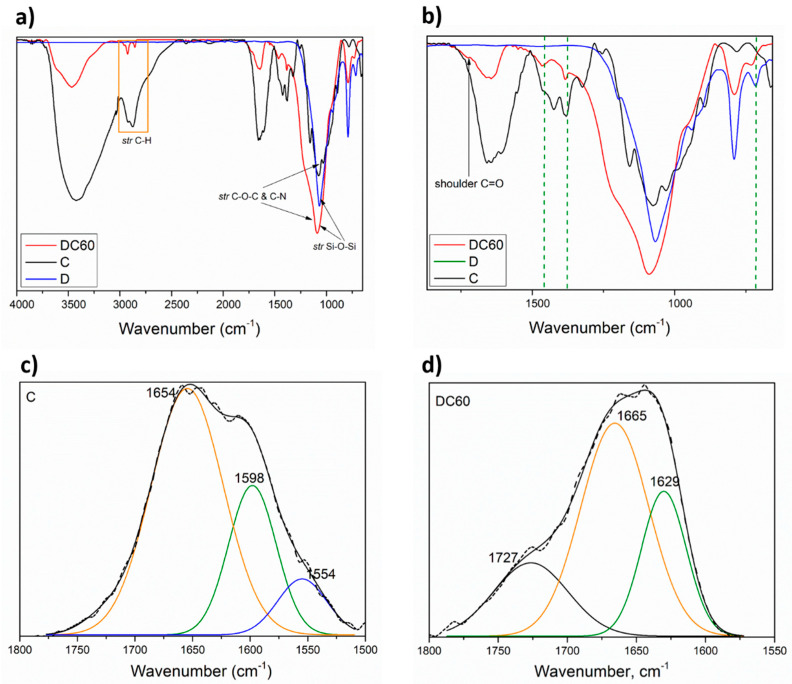
(**a**) FTIR of C, D and DC60 powders. (**b**) Selected wavenumber range, 600–2000 cm^−1^, for C, D and DC60 powders. (**c**) FTIR deconvolution of C sample in the wavenumber range, 1500–1800 cm^−1^. (**d**) FTIR deconvolution of DC60 sample in the wavenumber range, 1550–1800 cm^−1^.

The FTIR spectrum of D powder highlighted a strong peak correlated to the asymmetric stretching vibration of Si–O–Si (tetrahedral structure of (SiO_4_)^−4^) around 1070 cm^−1^ and a small peak around 790 cm^−1^ due to the symmetric stretching of Si–O–Si [9]; furthermore, a peak related to the silanol group (Si–OH) at ~723 cm^−1^ was also detected. In the FTIR spectrum of DCs powder (see the spectrum related to the DC60 sample in Figure 1a,b), the vibration peaks related to presence of both D and C vibration peaks were observed. In particular, the DC60 sample highlighted the multiple overlapping peaks due to OH and the N–H in the range 3200–3770 cm^−1^ wavenumber [12,33,34], the stretching vibration of C–H bonds in the range 2900–3000 cm^−1^. Furthermore, in the range 1800–1550 (see the deconvolution of FTIR spectrum in Figure 1d) the deformation vibration of the double peak of primary amine, *δ*–NH_2_, and the amide II, NH (a band of amide I centered at 1629 cm^−1^ appeared [35], of chitosan disappeared due to the hydrogen interactions that can be occur between the –NH_2_ an NH with the OH groups (silanol) and oxygen bridges of siloxane groups (−Si–O–Si-bridges), which act as adsorption sites located on the diatomite surface [36,37], above all because the production of the DCs was obtained by mixing in diatomite with solubilized chitosan in acetic water solution.

Furthermore, a shoulder around 1727 cm^−1^ correlated to the free carbonyl group. We hypothesized that free C=O carbonyls are residual groups that come from the reaction between the chitosan with acetic acid (−NH_2_ + CH_3_COOH = −NH_3_^+^CH_3_COO^−^) [38].

**Figure 2:**
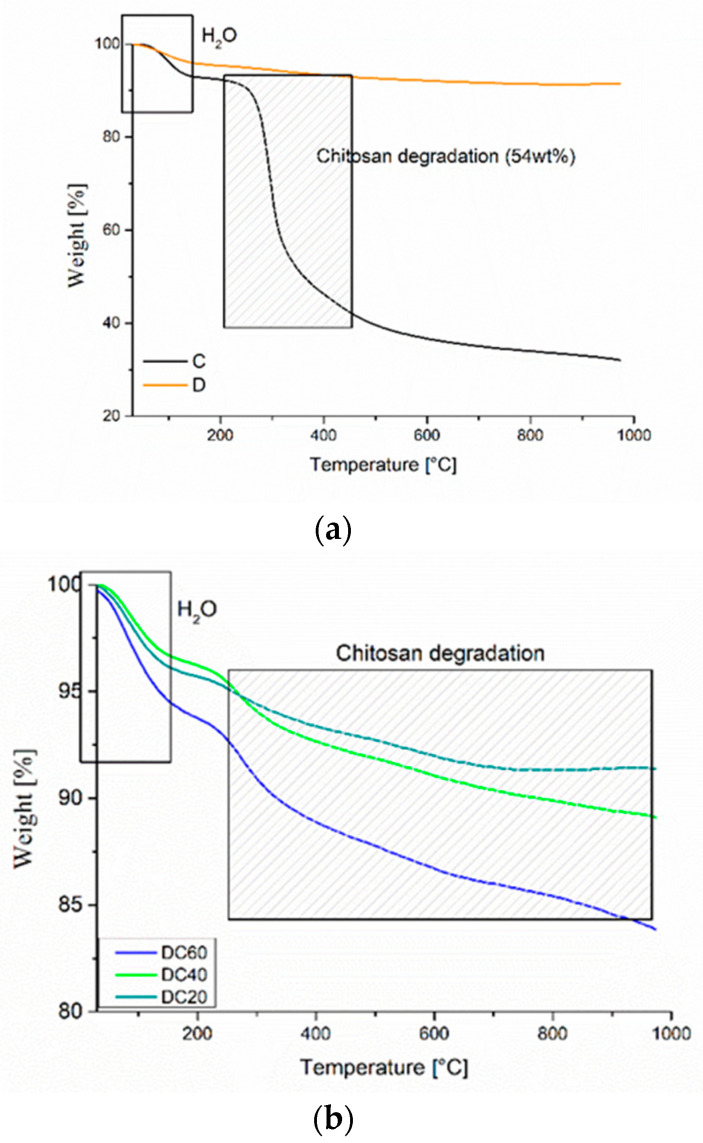
TGA of (**a**) C, D and (**b**) diatomite chitosans (DCs) powders.

The thermogravimetric analysis (TGA) investigation on the effect of pristine powders (C and D) on the hybrid powders (DC20, DC40 and DC60) are reported in Figure 2a,b. C sample highlighted two degradation stages related to (1) the water evaporation (~7 wt%) around 100 °C and (2) the decomposition of chitosan’s main chain (~54 wt%) in the range 250–400 °C [39]. For the TGA thermogram of D sample, only the water evaporation (~3 wt%) correlated to the humidity of the powder was observed. Finally, the thermograms of DCs (DC20, DC40 and DC60) powders highlighted the degradation steps related to both components: chitosan (depending on its percentage) and diatomite. The DCs samples depending on chitosan percentage would present degradation steps from about 30% to 10% (from DC60 to DC20, respectively). The real amount detected was lower, due to the chemico-physical interaction between chitosan and diatomite-based structure, as evidenced by FTIR spectra.

**Figure 3:**
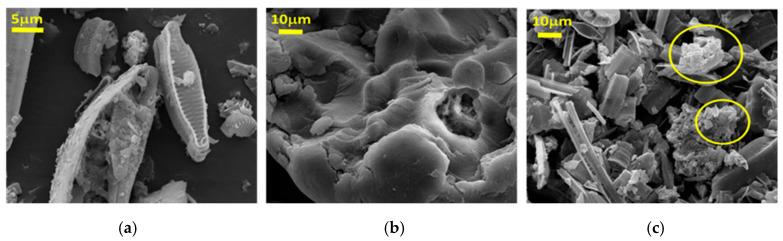
SEM microstructure (25.0 kV, magnification 775×) of (**a**) diatomite, (**b**) chitosan and (**c**) chitosan-modified diatomite.

Morphology of the samples is reported in Figure 3. The typical microstructure of frustules [29] with nanoporous channels was observed (Figure 3a), while chitosan showed its typical amorphous structure (Figure 3b) [39]. In DC60 sample (Figure 3c), the presence of diatomite frustules embedded by the chitosan matrix was observed (see in Figure 3c the part circled in yellow).

The selected PDCs hybrid (PDC60) was also characterized and compared with pristine PD sample.

**Figure 4:**
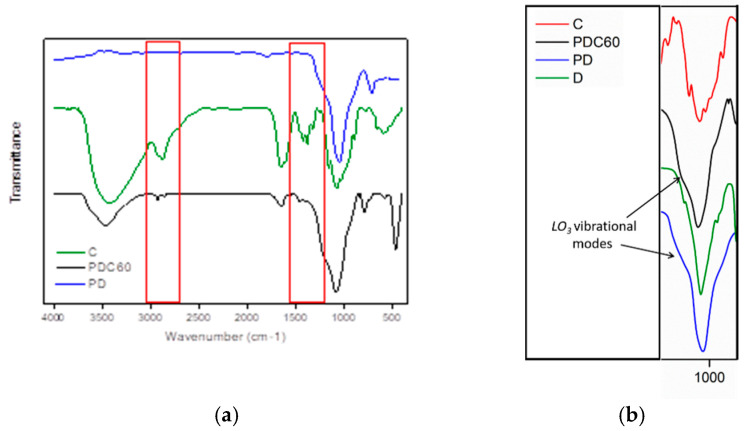
(**a**) FTIR spectra of porous diatomite (PD), C and PDC60. (**b**). FTIR spectra of PD and PDC60 in the range 900–1200 cm^−1^.

In Figure 4a, the FTIR spectra related to C, PD and PDC60 are reported. As observed, generally, the PD and PDC60 spectra highlighted the characteristic peaks of their main components (diatomite and chitosan), which indicates that chitosan was well embedded on diatomite structure. In addition, in Figure 4b) the FTIR spectra of PD and PDC60 in the range 900–1200 cm^−1^ are analyzed and compared with their constituent, D and C, in the 900–1200 cm^−1^. It is possible to observe that (as aforementioned reported) the D sample showed a broad absorption peak centered at 1070 cm^−1^ frequency that was assigned to the Si–O–Si asymmetric stretching peaks typical of silicate species [9,31,32]. The PD and PDC60 systems evidenced, in addition to the main band centered at 1050 cm^−1^ for the PD and 1081 cm^−1^ for PDC60, a further shoulder located around 1215 cm^−1^ ascribed to specific vibrational modes (namely, LO_3_ modes: Si–O–Si asymmetric stretching [9,40] means that the structure is highly crosslinked [40,41]).

**Figure 5:**
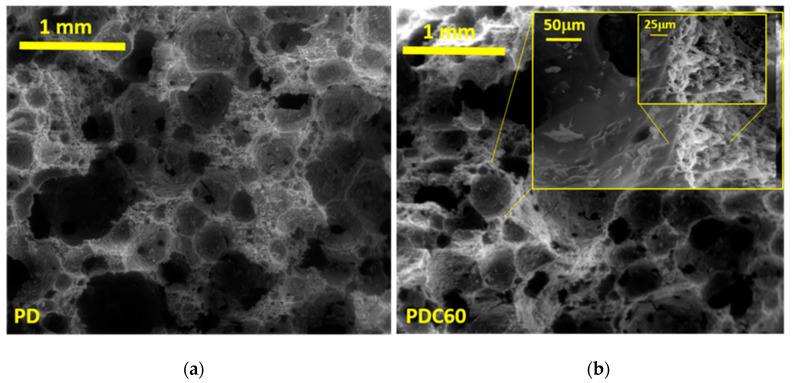
SEM image of (**a**) PD (20 kV, magnification 80×) and (**b**) PDC60 (inset of different magnification to highlight the presence of chitosan: 20 kV and 80×, 800× and 1600× for 1 mm, 50 μm and 25 μm respectively).

Thus, the effect of the chitosan–diatomite bond on the microstructure and the pore size distribution of produced hybrid foams was investigated and the SEM image of PD and PDC60 (at different magnifications) are shown in Figure 5a,b respectively). By comparing the SEM of PD and PDC60 at low magnification (80×), it is possible to observe that the chitosan did not affect the morphological structure of the produced foams. Furthermore, the PDC60 sample at high magnification (see the inset picture in Figure 5b) highlighted, mainly, a closed–open cells structure [42] with a large pore size distribution (400–100 µm), in addition in the cell-walls of the foam a nanoporosity, subjected to the intrinsic nanoporous structure of diatomite.

**Figure 6:**
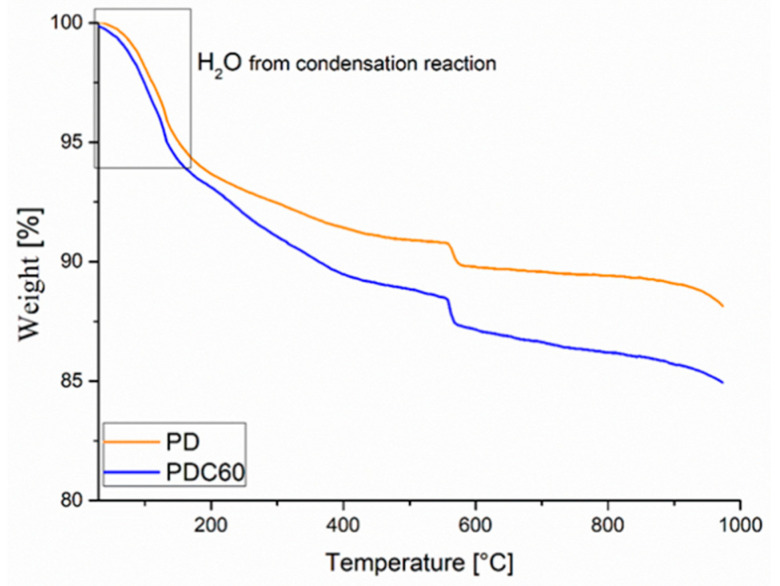
PD and PDC60 thermograms.

The PDC60 was also characterized through TGA analysis, and the thermogram is reported in Figure 6 and compared with pristine PD. As observed, both samples showed three degradation steps: the first occurs in the range 50–200° with weight loss of 5–6 wt% can be correlated to the consequence of the removal of the water comes from the condensation reactions of the sodium silicate solution [7,8,31]. The second and the third steps are attributed to the thermal degradation of the sodium hexafluorosilicate (catalyst), for instance, the first degradation of unreacted sodium hexafluorosilicate and the third (higher) weight loss to the vaporization of melted NaF [42,43], as well described in Galzerano et al. [9]. Furthermore, for the PDC60 in the range 250–400 °C, the chitosan degradation was also observed.

The density and porosity of PDC60 were also evaluated to verify the effect of chitosan on the physical properties of the foams. As shown in Table 1, the presence of chitosan into the matrix did not affect significantly the density and the porosity of the structure with respect to the pristine PD [27,42]. Moreover, the water absorption (that is directly correlated to the presence of open pores) and the high value of open porosity suggested that the PDC60 is suitable to be applied as an adsorbent for removing toxic compounds from aqueous media, such as the anionic dye Indigo Carmine [44].

**Figure 7:**
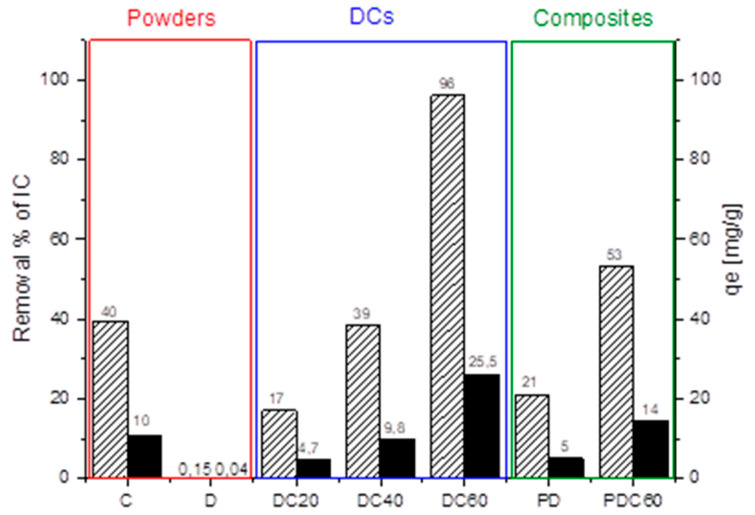
Removal percentage (dashed) and uptake capacity (black) of Indigo Carmine (IC) from water due to: raw materials (C and D), diatomite–chitosan-based powders (DCs) and porous materials (PD and PDC60).

The results of adsorption test are reported in Figure 7. The pristine D and C powders highlighted a different affinity towards the anionic dye: in particular, C showed a good interaction thanks to the electrostatic attraction between protonated amine group −NH^3+^ and anionic sulfonate group SO^3−^ group of the dye molecule; on the contrary, no absorption was recorded for D powder due to its anionic nature.

For this reason, the DCs hybrid showed a good absorption capacity towards IC dye from water. Its value is higher with respect to that of pristine C, probably due to a synergistic contribution of functional groups of C and to a larger specific area available in presence of diatomite. In addition, the removal efficiency of IC dye increased with the amount of C. This is likely due to the effect either of the presence of micropores on the diatomite surface [42,45] and of the chitosan [27] that, in the DCs hybrid, is embedded on the surface of D and, thus, shows a higher specific surface area available to interact with IC with respect to the raw powders.

The positive effect on the IC dye removal efficiency was also observed for the porous hybrid materials, PDCs. PDC60 sample showed a higher capacity of dye removal (about three times higher) with respect to pristine PD. This can be correlated to the synergistic effects that occurred in the hybrid foam, namely, the interaction of functional groups of C with IC and the higher surface area able to increase the interaction.

Actually, the aim is assessing the possibility to produce, through a combination of several manufacturing processes, an adsorbent material either in powder or in monolithic form useful in different fields. Depending on the specific application (continuous or batch removal process), it is possible to select the best “shape” of the adsorbent. So, even if the PCDs production can appear to be an expensive procedure, it represents the best solution in fixed-bed columns where a selfsupporting adsorbent is needed. It will be interesting to work on the following two items: firstly the regeneration/reutilization of the saturated adsorbent and secondly testing these innovative adsorbents in a real polluted scenario.

## 4. Conclusions

In the present work, we have designed, synthesized and characterized potential absorbent materials based on diatomite–chitosan modified hybrid powders. The interaction between diatomite and chitosan was confirmed by FTIR data, which allowed one to hypothesize interactions that occurred between the hydrogen and the functional groups (mainly, the −NH_2_ groups) of chitosan with the adsorption site groups of silanol located on the diatomite surface. The hybrid powders were used to produce porous diatomite–chitosan hybrids as potential absorbents to be applied in an environmental scenario. In particular, the porous hybrids can be produced and used in the form of both powder (type activated carbon filter) and monolith (self-supporting); this allows one to apply the novel porous hybrids in several application fields. The hybrid powders were used to produce porous diatomite–chitosan hybrids. 

The typologies of materials, powders and foams were preliminary tested to evaluate their efficiency for dye-IC adsorption. The removal efficiency of the hybrid powders (DCs) and hybrid porous materials (PDCs) in the adsorption of IC from a water solution was tested. Results highlight that the presence of chitosan affects significantly and positively the adsorption capacity of both systems with respect to the raw materials and the pristine porous materials; furthermore, it has been shown that the dye uptake increases as the amount of C into the diatomite hybrids increases. These preliminary results suggest that both of the produced materials can potentially be promising adsorbents for the removal of anionic dyes from wastewater.

## Figures and Tables

**Table 1:** Apparent density (ρ_app_), open porosity (OP) and closed porosity (CP) and water adsorption capacity (WA) for PDC60.

Hybrids	ρ_app_ kg/m^3^	OP%	CP%	WA%
PD	502 ± 30	58.97 ± 5	22.38 ± 2	124.97 ± 20
PDC60	533 ± 15	57.73 ± 7	19.04 ± 3	118.15 ± 25
